# Supplementary material for: Cryopreservation of Pig Semen Using a Quercetin-Supplemented Freezing Extender
Source: Life (Basel). 2022 Jul 29;12(8):1155. doi: 10.3390/life12081155 (PMC9410179; doi:10.3390/life12081155)
Supplement: Supplementary file 1 [file life-12-01155-s001.zip › life-1825003-supplementary.pdf]

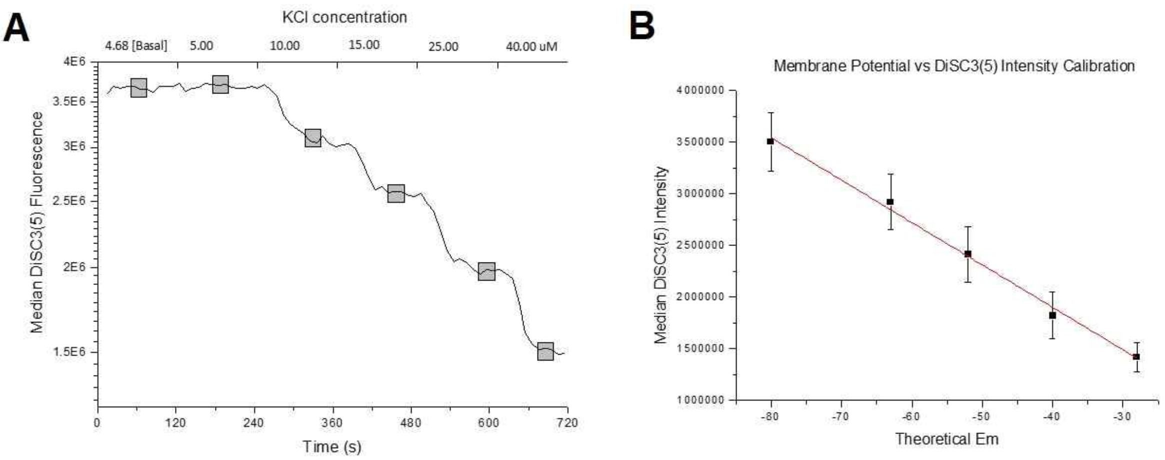

Supplemental Figure S1. Measurement of Absolute Membrane Potential of Quercetin Treated Porcine Spermatozoa. The temporal data acquisition of sperm samples using flow cytometry. (A) Grey boxes of the plot depict the plateau fluorescence region corresponding to each KCl concentration. (B) Theoretical absolute membrane potential versus DiSC3(5) fluorescence intensity calibration linear model is shown in figure 2 (R2=0.9799, equation: y= -41101.46x + 254879.32).
